# The anti-obesity effects of rhein on improving insulin resistance (IR) and blood lipid levels are involved in endoplasmic reticulum stress (ERs), inflammation, and oxidative stress in vivo and vitro: The protective mechanism of rhein in preventing obesity

**DOI:** 10.1080/21655979.2021.1969196

**Published:** 2021-09-13

**Authors:** Li Ji, Huan Gu

**Affiliations:** a Department of Pediatrics, Guang’anmen Hospital, Chinese Academy of Traditional Chinese Medicine, BeiJing, China; b Department of Cardiology of Integrated Traditional Chinese and Western Medicine China-Japan Friendship Hospital, BeiJing, China

**Keywords:** Rhein, obesity, endoplasmic reticulum stress, inflammation, oxidative stress, apoptosis

## Abstract

Rhein extensive biological effects including anti-inflammatory, antioxidant stress, and improving glucose and lipid metabolism. In the present study, the effects of rhein were examined on endoplasmic reticulum stress (ERs) and inflammation in obesity-induced rats. SD rats were fed with a normal diet or a high-fat diet. Meanwhile, rats fed with high-fat diet were also administrated with different doses of rhein for 6 weeks. The pathologic changes of pathoaorta pectoralis were evaluated using hematoxyline eosin (HE) strain, and cell apoptosis levels were investigated using TUNEL staining and flow cytometry. We also performed p62 immunofluorescent staining in 3T3-L1 cells. In the present study, we found that rhein administration exerted inhibitory effects on weight, inflammatory factor levels, and oxidative stress. Meanwhile, insulin resistance (IR), blood lipid levels and pathological injury of aorta pectoralis were also improved by rhein administration. Besides, rhein also affected ERs in peripheral blood and adipose tissue *in vivo*. Moreover, rhein significantly reduced cell apoptosis in aorta pectoralis and adipose tissue in vivo. According to oil red staining, adipogenic differentiation was decreased by rhein treatment in vitro. Immunofluorescence staining of p62 showed that rhein contributed to a significant increase in p62 expression in vitro. In addition, rhein treatment significantly decreased peroxisome proliferators-activated receptor (PPAR)γ levels and upregulated insulin receptor (INSR) in vitro. In summary, the anti-obesity effects of rhein were considered to be related with the involvement of ERs, inflammation, oxidative stress, PPARγ, and INSR.

## Introduction

With the continuous improvement of people’s living standards, the number of obese people is increasing year by year and the trend is getting younger. Obesity is often accompanied by serious complications, such as hypertension, hyperlipidemia, and diabetes [1,2]. Accumulating studies have shown that rhein has a clear effect on lowering blood lipids. At the same time, it also has anti-inflammatory, antioxidant stress, anti-tumor, improve glucose, lipid metabolism, and insulin resistance [3–9]. Additionally, rhein inhibited cell proliferation, the production of adenosine triphosphate, and mitochondrial transmembrane potential in tumor cells but promoted apoptosis, the levels of glucose regulatory protein 78(GRP78) and activating transcription factor 6 (ATF6) [10,11]. Previous studies also demonstrated that rhein could inhibit the up-regulation of C/EBP homologous protein (CHOP) gene expression in adipocytes and suppress the proliferation, differentiation, and secretion function of human precursor adipocytes by affecting PPARγ [12,13].

Endoplasmic reticulum stress and oxidative stress are one of the important mechanisms for upstream induction and expansion of inflammatory response [14]. The immune mechanism of children is not yet mature. Long-term chronic inflammatory response can lead to abnormal proliferation, differentiation, and secretion of adipocytes [15,16]. Meanwhile, inflammatory response is also an important mechanism causing insulin resistance, which may be related to inflammatory factors acting on INSR to change the phosphorylation site and process, and eventually lead to insulin resistance [17,18]. The predicted targets of rhein by BATMAN database also found that rhein can directly regulate Interleukin (IL-1)β and IL-13 which possess strong pro-inflammatory effects.

Altogether, the protective effects of rhein against obesity could appear to rely on the regulation of ERs, autophagy, and inflammation, possibly also by PPARγ and INSR. HFD could lead to abnormal weight gain and lipid deposition [19]. In the present study, the rats were fed with HFD to induce an obesity model of impuberism female rat. By observing the effects of rhein on juvenile rat obesity model and comparing the results in related animal and cell experiments, we analyzed the internal mechanism of rhein, which laid experimental foundation for traditional Chinese medicine in the treatment of childhood obesity.

## Method

Animal

SPF grade female SD rats (20 days), with body weight of 67–76 g, were purchased from Beijing Vital River Laboratory Animal Technology Co., Ltd (Beijing, China). Rats, which were accessible to food and water ad libitum, were exposed to light alternately day and night for 12 hours at a temperature of 20 ~ 25°C and a relative humidity of 50%–70%. Rats were fed adaptively for 1 week and the experiment lasted for 6 weeks. During the period, the rats were administrated rhein (being suspended in 0.5% sodium carboxymethyl cellulose solution) or equal volume of sodium carboxymethyl cellulose solution (control) intragastrically once a day for 6 weeks. The weight of rats was recorded every week. We observed the basic physiological state of rats during feeding period and judged whether rhein produced possible side effects. Animal experiments were approved by the Animal Ethics Committee of China–Japan Friendship Hospital. Fast blood glucose levels were detected from tail of rats by a blood glucose meter (Roche Diabetes Care GmbH). 5 mL of blood that was collected from abdominal aorta was centrifugated to obtain serum and stored at −20°C. The rats were sacrificed by cervical dislocation to collect adipose tissue and thoracic aorta. Body length is measured by measuring the distance from nose tip to anus. Lee’s index = (weight×10^3^/ body length) [20]. The rats were divided into five groups: normal chow, high-fat diet (HFD) (D12492, 60% kcal%, Research Diets, USA), high-fat diet +Rhein-L (lose dose, 20 mg/kg), high-fat diet (60% kcal%) + Rhein-M (medium dose, 40 mg/kg), high-fat diet (60% kcal%) +Rhein-H (high dose, 80 mg/kg).

The detection of total cholesterol (TC), triglyceride (TG), high-density lipoprotein (HDL), low density lipoprotein (LDL) and insulin

According to the instructions in the reagent kits, the contents of TC, TG, HDL, and LDL in serum were determined (Nanjing Jiancheng Bioengineering Institute, Nanjing, China). The insulin in serum was detected through ELISA kit in accordance with manufacturer’s protocol (Wuhan ColorfulGene Biological Technology Co.,LTD, Wuhan, China).

The detection of inflammation-related factors and oxidative stress-related factors

Serum IL-6, IL-1β, and tumor necrosis factor (TNF-α) levels were detected by enzyme-linked immunosorbent assay (ELISA) in accordance with the kit instructions (mlbio, Shanghai, China). The ROS levels, malondialdehyde (MDA) and SOD activities were detected using corresponding kits following manufacturer’s instructions (Beyotime, Shanghai, China).

HE staining

The rats were sacrificed by cervical dislocation. Macrovascular tissue was collected and fixed in 4% paraformaldehyde solution and the tissues were dehydrated, sectioned, dewaxed, hydrated, and stained. Then, HE staining was performed according to manufacturer’s protocol (solarbio, Beijing, China). The pathological changes of liver adipose tissue of rats in each group were observed under a microscope (Olympus).

Tunel assay

The paraffin sections of macrovascular tissue and adipose tissue were prepared. After xylene dewaxing, the sections were washed with PBS twice and incubated with TUNEL solution at 37°C for 60 min (Beyotime, Shanghai, China) following manufacturer’s guidance. Blocked with anti- fluorescence quenching solution, the sections were subsequently observed under a fluorescence microscope (Olympus).

Western blot

50 μL RIPA protein lysate containing protease inhibitor was added into the collected adipose tissue. The above mixture was centrifuged at 12,000 r/min for 20 min, and then the supernatant was collected. The protein concentration was quantified using BCA kits. The protein loading buffer solution was added and heated at 95°C for 10 min to denature the protein. The proteins were separated by 12% sodium dodecyl sulfate-polyacrylamide gel electrophoresis and transferred to PVDF membranes. Blocked with 5% skim milk for 2 h, the PVDF membranes were incubated with the primary antibodies overnight at 4°C. On the next day, the proteins were incubated with secondary antibodies at 1 h at room temperature. The Odyssey imaging system was used for scanning and Image J analysis software (National Institutes of Health) was used for analysis.

Cell culture

3T3-L1 preadipocytes were seeded into 24-well plates and cultured in Preadipocyte Differentiation Medium (ScienCell Research Laboratories, USA) which could induce adipogenic differentiation, at 37°C with 5% CO_2_. Raw246.7 was cultured in 1640 medium containing 10% FBS at 37°C with 5% CO_2_. The supernatant was collected and used to incubate with 3T3-L1 preadipocytes after Raw246.7 cells were stimulated with lipopolysaccharide (LPS) (1 µg/mL). In rhein treated group, different concentrations of rhein (low, medium, high: 50 µM, 75 µM, and 100 µM) were used to treat cells.

Oil red staining

The accumulation of lipid drops was detected with oil red staining. 3T3-L1 cells were fixed using 4% paraformaldehyde and then washed with PBS for 3 times. Then, cells were incubated with oil red O solution (Shanghai regal Biology Technology Co, Ltd, Shanghai, China) for 1 h at room temperature. The liquid was removed and 1 mL isopropanol was added to extract the fat droplets in the adipocytes. After 20 min, the absorbance at 550 nm was detected using a microplate reader (Thermo Fisher Scientific).

Flow cytometry

Cells were collected in a density of 1 × 106/mL, centrifuged at 500–1000 r/min for 5 min and fixed by 70% pre-cooled ethyl alcohol (Solarbio, Beijing, China) for 2 h at 4°C. After resuspension in 3 ml PBS for 5 min, the cells were filtered by a cell strainer and stained with 1 ml of Propidium Iodide staining solution (Yeasen Biotechnology, Shanghai, China) in dark for 30 min at 4°C. The absorbance was measured by BriCyte E6 flow cytometer (Mindray, Shenzhen, China).

Immunofluorescence assay

3T3-L1 cells were fixed with methanol and permeabilized with 0.1% Triton X-100 for 5 min. Subsequently, the cells were incubated with anti-p62 (ab109012, Abcam, England) after incubation with 10% normal goat serum for 1 h, followed by a further incubation at room temperature for 1 h with a goat secondary antibody to rabbit IgG (ab150081, Abcam, England). Nuclear DNA was labeled in blue with DAPI. Image was taken with a fluorescence microscope (Olympus).

Statistical analysis

Prisma 8.0 software was used for statistical analysis of the experimental data and the results were expressed as mean± standard deviation (SD). One-way analysis of variance was used for comparison among multiple groups, followed by Turkey’s test.

## Result

### Rhein inhibited excessive weight gain in rats fed with high fat

**Figure 1.**
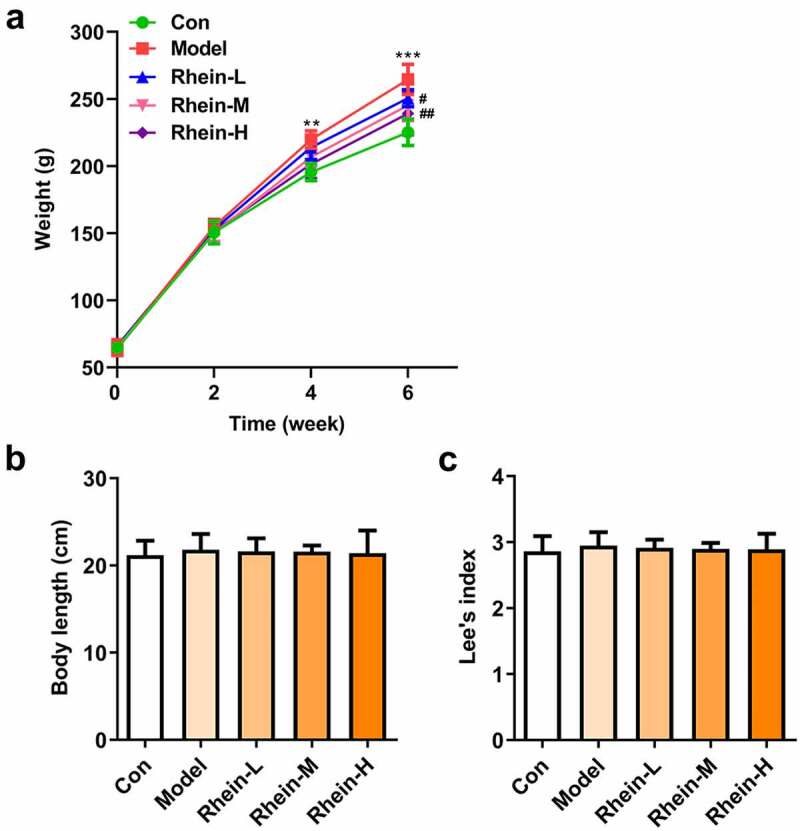
Effects of rhein on body weight and size of rat. The rats of different groups received the following treatment for six weeks, respectively. normal chow, high-fat diet (HFD) (D12492, 60% kcal%, research diets, USA), high-fat diet +Rhein-L (lose dose), high-fat diet (60% kcal%) + Rhein-M (medium dose), high-fat diet (60% kcal%) +Rhein-H (high dose). (a) Growth curve of mice in each group. (b) The body length after rhein administration for 6 weeks. (c) Lee’s index. n = 6 rats/group. HFD, high-fat diet-fed rats. **p < 0.01, ***p < 0.001 versus control. #p < 0.05, ##p < 0.01 versus HFD

The body weight index is the most direct embodiment of whether rhein can prevent obesity in rats. The weight changes of rats during feeding period are shown in Figure 1a. At the beginning of the experiment (week 0), there was no difference in body weight among the five groups. After 6 weeks of feeding, the body weight of the HFD-fed group was significantly higher than normal feed group. Rhein administration effectively inhibited the excessive increase in weight induced by HFD in a dose-dependent manner (Figure 1a). However, there was no significant difference of body length and lee’s index among different groups (Figure 1b and c).

## Rhein-reduced serum lipid level in rat fed with high fat

**Figure 2.**
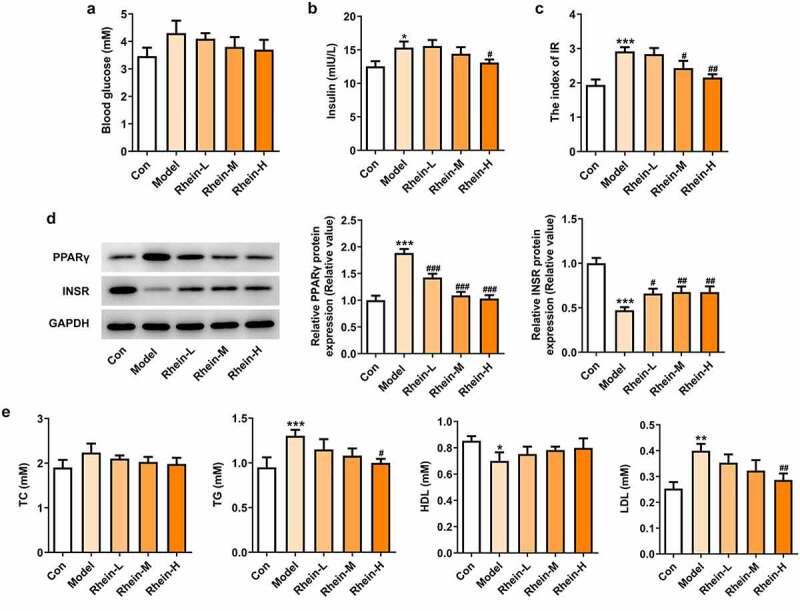
Effects of rhein on IR and blood lipid levels. The rats of different groups were fed by the following administration, respectively, for six weeks, respectively: normal chow, high-fat diet (HFD) (D12492, 60% kcal%, Research Diets, USA), high-fat diet +Rhein-L (lose dose), high-fat diet (60% kcal%) + Rhein-M (medium dose), high-fat diet (60% kcal%) +Rhein-H (high dose). (a) The blood glucose. (b) Insulin levels. (c) Insulin resistance levels. (d) Western blotting analysis of PPARγ and INSR. n = 6 rats/group. HFD, high-fat diet-fed rats. (e) The blood lipid levels. *p < 0.05, **p < 0.01, ***p < 0.001 versus control. #p < 0.05, ##p < 0.01, ###p < 0.001 versus HFD

Next, the effect of rhein on serum lipid level was examined. As shown in Figure 2. There were different differences among different groups (Figure 2a). Compared with the control group, HFD-induced rats displayed an increase in insulin levels and IR that was then reversed by rhein with high dose (Figure 2b). Additionally, rhein in medium dose could also reduce IR (Figure 2c). The levels of PPARγ were significantly enhanced in rats fed with HFD in comparison with normal chow rats, while the levels of INSR were markedly reduced (Figure 2d). Rhein treatment reversed the elevated expressions of these proteins in a concentration-dependent manner in contrast with Model rats. The accumulation of excess fat in obese individuals often leads to elevated levels of lipids (including triglycerides and cholesterol) in the blood. The serum biochemical index (TG, TC, LDL and HDL) of rat in each group was further analyzed. In addition, the contents of TG and LDL-C FFA in serum of rats in Model group gained a huge growth while HDL was significantly decreased (Figure 2e). However, there was no significant change in TC levels (Figure 2e). The above results revealed that rhein administration can improve the dyslipidemia caused by HFD.

## The elevated inflammation and oxidative stress levels were reduced by rhein

**Figure 3.**
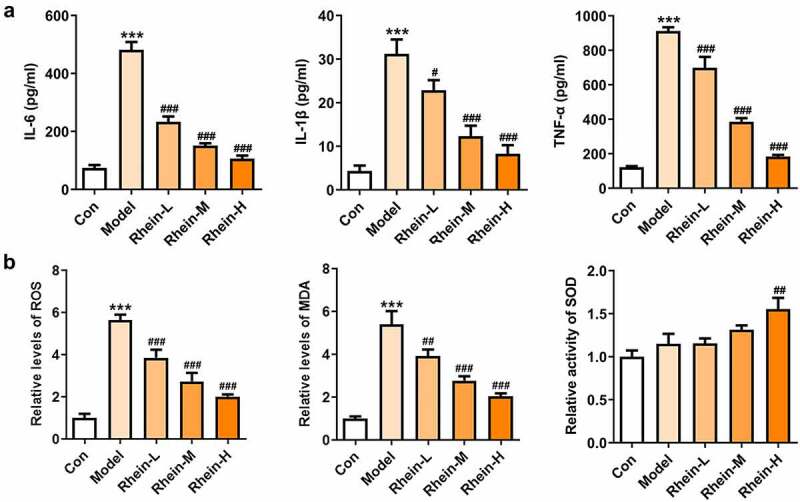
Effects of rhein on inflammation and oxidative stress. The rats of different groups were administrated through the following treatment for six weeks, respectively. normal chow, high-fat diet (HFD) (D12492, 60% kcal%, Research Diets, USA), high-fat diet +Rhein-L (lose dose), high-fat diet (60% kcal%) + Rhein-M (medium dose), high-fat diet (60% kcal%) +Rhein-H (high dose). (a) The detection of inflammatory markers levels. (b) Oxidative stress levels. n = 6 rats/group. HFD, high-fat diet-fed rats. ***p < 0.001 versus control. #p < 0.05, ##p < 0.01, ###p < 0.001 versus HFD

**Figure 4.**
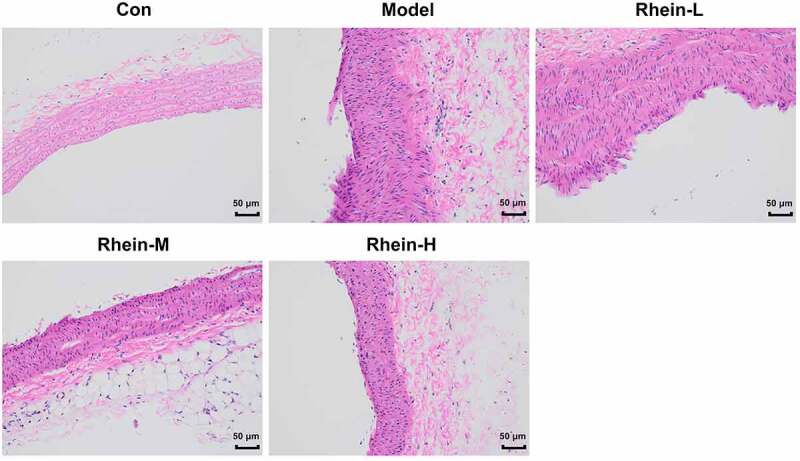
The effects of rhein on pathological changes of blood vessel were evaluated by HE staining. After the rats of different groups were fed by the following administration, respectively, for six weeks, respectively: normal chow, high-fat diet (HFD) (D12492, 60% kcal%, Research Diets, USA), high-fat diet +Rhein-L (lose dose), high-fat diet (60% kcal%) + Rhein-M (medium dose), high-fat diet (60% kcal%) +Rhein-H (high dose), blood vessel tissue was collected

To examine whether rhein was involved in the inflammation and oxidative stress induced by rhein, the expression levels of related markers were detected and the pathology changes were analyzed by HE staining. The expression levels of inflammation-related factors, including IL-6, IL-1β, and TNF-α were simultaneously elevated in the peripheral blood of rats fed with high fat (Figure 3a). However, the elevations in the peripheral blood were gradually reduced by rhein with the increase of dose. We further found that the levels of ROS and MDA showed an increase in rats administrated with HFD while SOD levels displayed a reduction as relative to control group (Figure 3b). The HE staining of aorta pectoralis demonstrated that the lumen was smooth, endothelial cells, internal and external elastic layer were continuous and complete, and smooth muscle cells were orderly arranged. In HFD-induced group, the vascular lumen was rough, the endothelial cells of the large vessels were damaged and the continuity of the endothelial cells was destroyed. Additionally, there was infiltration in inflammatory cells. These pathological injuries were greatly improved by rhein administration in a dose-dependent manner (Figure 4).

## Vascular apoptosis and ER stress in rats fed with HFD were alleviated by rhein administration

**Figure 5.**
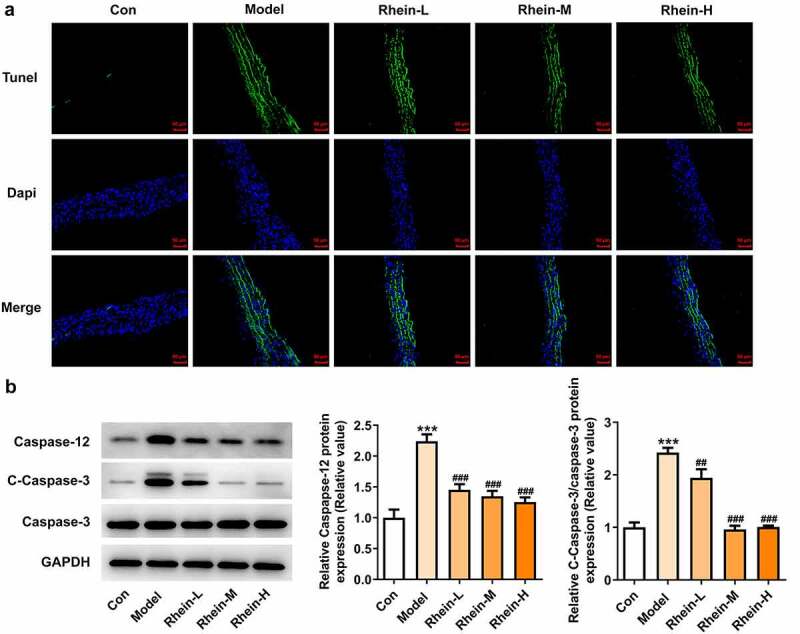
Rhein suppressed cell apoptosis of vascular tissues. The rats of different groups received the following treatment for six weeks, respectively. normal chow, high-fat diet (HFD) (D12492, 60% kcal%, Research Diets, USA), high-fat diet +Rhein-L (lose dose), high-fat diet (60% kcal%) + Rhein-M (medium dose), high-fat diet (60% kcal%) +Rhein-H (high dose). (a) Tunel analysis of vascular tissues. (b) Western blotting analysis of caspase12 and 3. n = 6 rats/group. HFD, high-fat diet-fed rats. ***p < 0.001 versus control. ##p < 0.01, ###p < 0.001 versus HFD

The increased apoptosis levels in vascular tissue in HFD-induced rats were found to be markedly decreased by rhein. Attempting to determine the effects of rhein on apoptosis pathway, we examined the expressions of key mediators involved in apoptosis induction such as caspase12, cleaved caspase3, and caspase3. The expressions of caspase12 and cleaved caspase3 in vascular tissues were much higher than those of the control group (Figure 5a, b). Moreover, the rhein concomitantly decreased the expressions of these two parameters.

**Figure 6.**
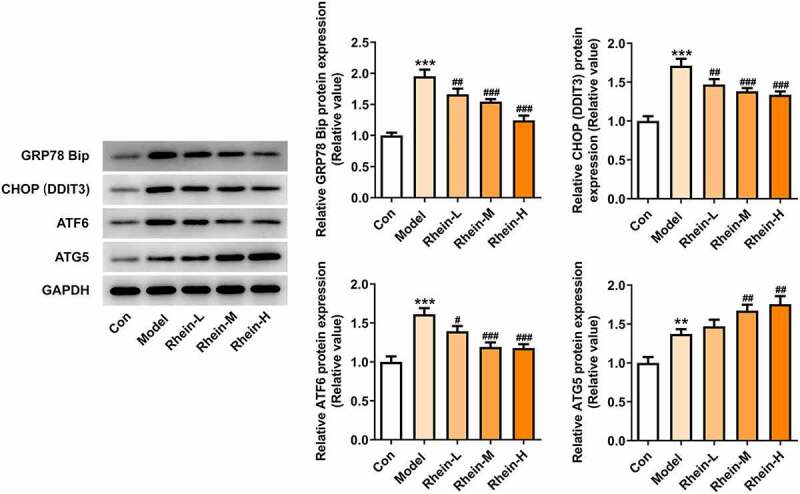
Rhein lowed the expression levels of ERs-related proteins and ATG5 levels. n = 6 rats/group. The rats of different groups received the following treatment for six weeks, respectively. normal chow, high-fat diet (HFD) (D12492, 60% kcal%, Research Diets, USA), high-fat diet +Rhein-L (lose dose), high-fat diet (60% kcal%) + Rhein-M (medium dose), high-fat diet (60% kcal%) +Rhein-H (high dose). HFD, high-fat diet-fed rats. ***p < 0.001 versus control. #p < 0.05, ##p < 0.01, ###p < 0.001 versus HFD

**Figure 7.**
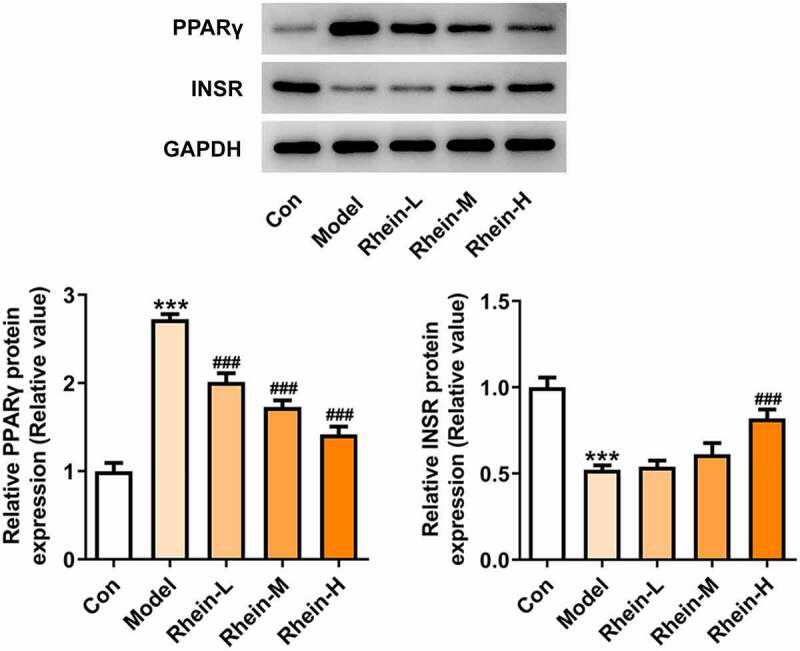
Effects of rhein on the expression levels of PPARγ and INSR through Western blotting analysis. n = 6 rats/group. The rats of different groups received the following treatment for six weeks, respectively. normal chow, high-fat diet (HFD) (D12492, 60% kcal%, Research Diets, USA), high-fat diet +Rhein-L (lose dose), high-fat diet (60% kcal%) + Rhein-M (medium dose), high-fat diet (60% kcal%) +Rhein-H (high dose). HFD, high-fat diet-fed rats. ***p < 0.001 versus control. ###p < 0.001 versus HFD

We found that the expressions of ERs-related proteins GRP78, CHOP and ATF6, and autophagy marker ATG5 were remarkably increased in the vascular tissues of HFD-induced rats compared to control rats, which were then reduced by rhein (Figure 6). In contrast, compared with rats fed with HFD, rhein further induced the increase of ATG5 level in vascular tissue. Some studies have revealed that PPARγ and INSR are implicated in IR. Increased PPARγ and decreased INSR in adipose tissue caused by high-fat diet were greatly reversed by rhein treatment (Figure 7).

**The increased inflammation and oxidative stress in adipose tissue of rats with HFD administration were improved by rhein**

**Figure 8.**
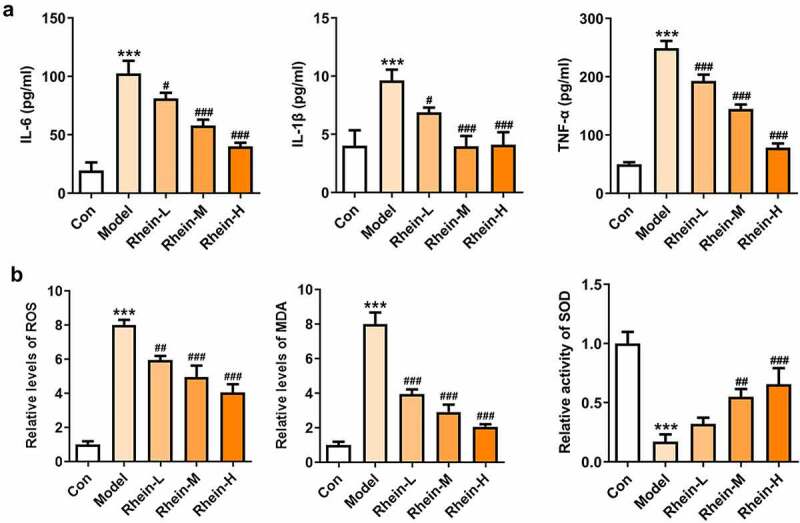
Effects of rhein on inflammation and oxidative stress in adipose tissue. The rats of different groups received the following treatment for six weeks, respectively. normal chow, high-fat diet (HFD) (D12492, 60% kcal%, Research Diets, USA), high-fat diet +Rhein-L (lose dose), high-fat diet (60% kcal%) + Rhein-M (medium dose), high-fat diet (60% kcal%) +Rhein-H (high dose). (a) The analysis of inflammatory markers levels. (b) Oxidative stress levels. n = 6 rats/group. HFD, high-fat diet-fed rats. ***p < 0.001 versus control. #p < 0.05, ##p < 0.01, ###p < 0.001 versus HFD

Studies have shown that inflammation of adipose tissue played a dominant role in the development of symptoms such as obesity and insulin resistance [21]. Adipose tissue participated in the regulation of fat and glucose homeostasis in the body [21]. It is noted that obese people have abnormal proliferation and hypertrophy of adipocytes, meanwhile, the levels of various inflammatory factors were increased in adipose tissue, thus causing chronic inflammation in the body, disrupting the insulin-signaling pathway in metabolism-related tissues, and reducing the insulin sensitivity of adipocytes [22–24]. The levels of related inflammatory cytokines such as IL-6, IL-1β and TNF-α were significantly increased in adipose tissue of HFD group compared with control group (Figure 8a). MDA was recognized to be as an indicator implying oxidative stress levels in rats with obesity fed with high fat diet [25,26]. By analyzing oxidative stress levels of adipose tissue, we noted that the expressions of ROS, MDA and SOD were significantly increased in HFD group when compared with control, and rhein had marked effects on reducing ROS and MDA levels and increasing ROS levels (Figure 8b).

## Adipose tissue apoptosis and ER stress in rats fed with HFD were alleviated by rhein administration

**Figure 9.**
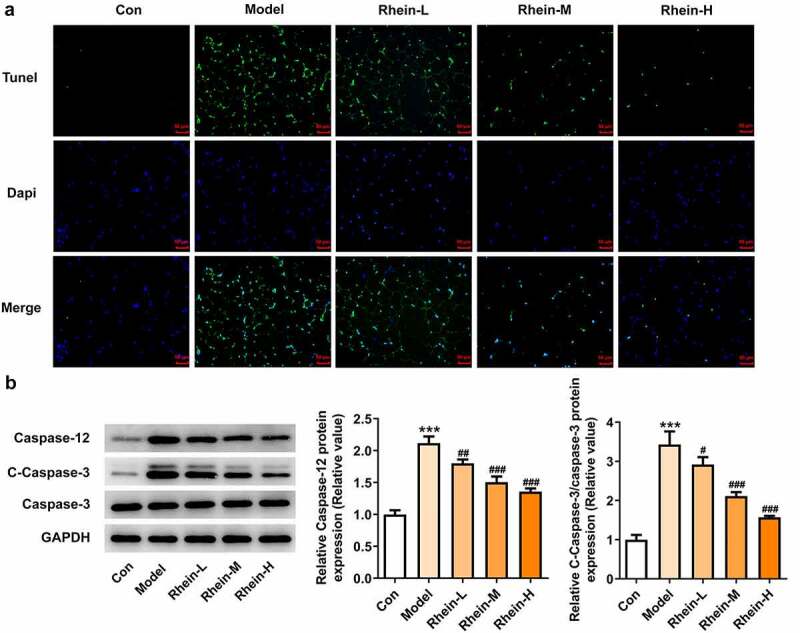
Effects of rhein on apoptosis and ER stress of adipose tissue in SD rats fed with HFD. The rats of different groups received the following treatment for six weeks, respectively. normal chow, high-fat diet (HFD) (D12492, 60% kcal%, Research Diets, USA), high-fat diet +Rhein-L (lose dose), high-fat diet (60% kcal%) + Rhein-M (medium dose), high-fat diet (60% kcal%) +Rhein-H (high dose). (a). Tunel staining for adipose tissue. (b). western blot analysis of adipose tissue showing the expression of caspase12, caspase3 and cleaved caspase3. n = 6 rats/group. HFD, high-fat diet-fed rats. ***p < 0.05 versus control. #p < 0.05, ##p < 0.01, ###p < 0.001 versus HFD

**Figure 10.**
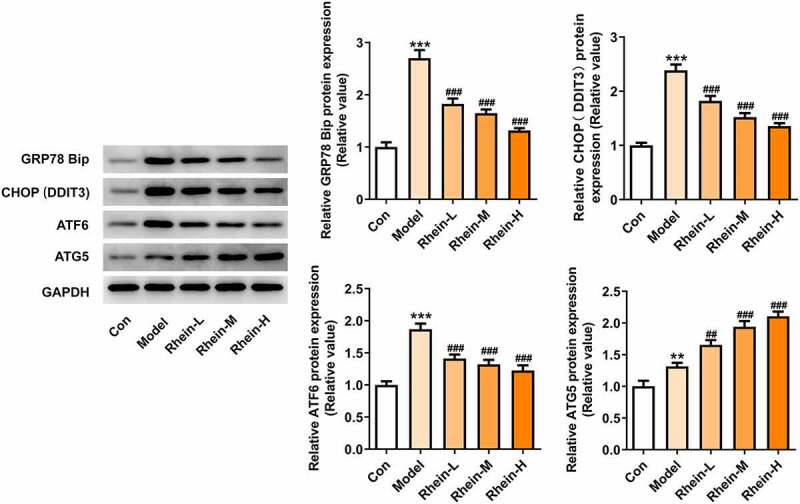
Effects of rhein on ER stress of adipose tissue in SD rats fed with HFD. western blot analysis of adipose tissue showing the expression of ERs-related protein and ATG5. The rats of different groups received the following treatment for six weeks, respectively. normal chow, high-fat diet (HFD) (D12492, 60% kcal%, Research Diets, USA), high-fat diet +Rhein-L (lose dose), high-fat diet (60% kcal%) + Rhein-M (medium dose), high-fat diet (60% kcal%) +Rhein-H (high dose). n = 6 rats/group. HFD, high-fat diet-fed rats. ***p < 0.05 versus control. ##p < 0.01, ###p < 0.001 versus HFD

We next attempted to assess the effects of rhein on apoptosis and ER stress in rats administrated with HFD. Compared with control group, the apoptosis levels of adipose tissue in HFD-induced rats gained a huge increase, which were subsequently decreased by rhein (Figure 9a). It was also found that the increased expressions of caspase12 and cleaved caspase3 in HFD-induced rats were diminished by rhein (Figure 9b). The induction of ERs often triggers autophagy [27]. To examine whether rhein affected ER stress of adipose tissue in HFD-induced rats, we measured the expressions of following ER stress markers: GRP78, CHOP and ATF6. As Figure 10 depicted, the increased expressions of GRP78, CHOP and ATF6 in rats fed with HFD were decreased after rhein treatment. Moreover, it is noted that the inhibitory effects of rhein on GRP78, CHOP and ATF6 expressions were in a concentration-dependent manner. We next assessed autophagy marker expression specifically in adipose tissue and found that the levels of ATG5 were higher in HFD-induced rats compared with control, which were further increased by rhein administration.

## Rhein treatment reduced lipid formation and apoptosis

First, we induced Raw246.7 macrophages with LPS (1 µg/mL) in vitro to collect the supernatants of the macrophages, and detect the levels of IL-6, IL-1β and TNF-α. Macrophages have a great influence on adipogenic differentiation, survival and proliferation of preadipocytes. Thereafter, 3T3-L1 preadipocytes were co-cultured with collected supernatant. We further determined whether the adipocytes adipogenesis was affected by rhein treatment.

**Figure 11.**
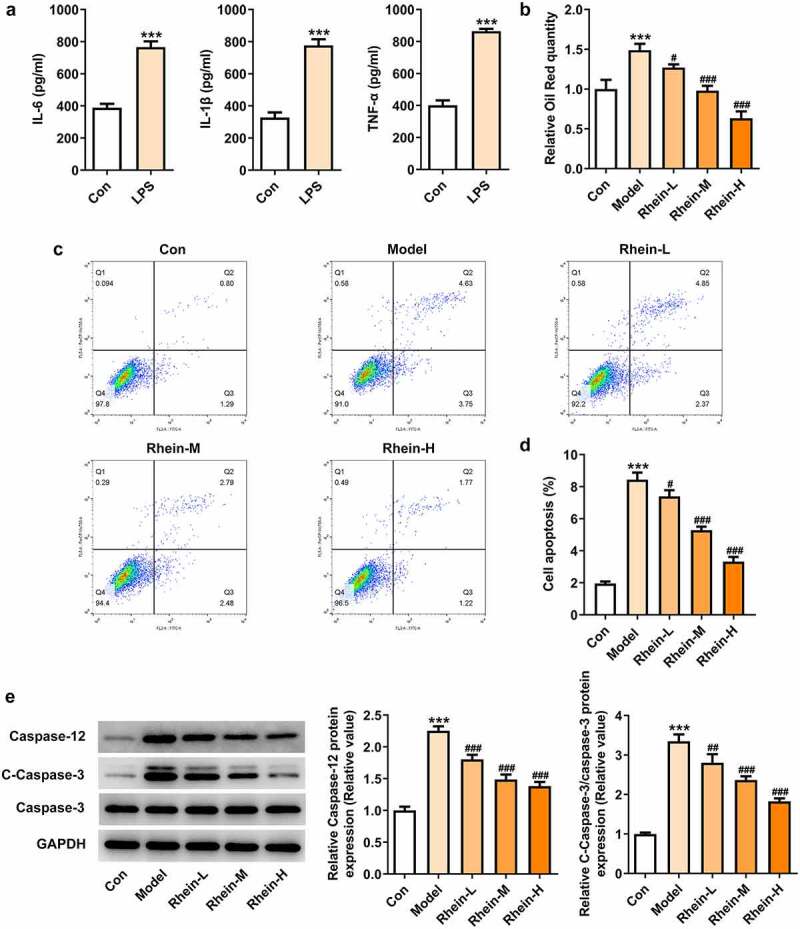
Rhein treatment decreased lipid formation and apoptosis of 3T3-L1 induced by inflammatory factors. The supernatant culturing Raw246.7 cells with LPS stimulation was collected and used to incubate with 3T3-L1 preadipocytes. (a). The levels of inflammatory factors, IL-6, IL-1β and TNF-α, in LPS-induced Raw246.7 macrophages. (b). Quantitative analysis of lipid droplet accumulation. (c, d). Detection of cell apoptosis by flow cytometry in 3T3-L1 cells. (e). Representative images of caspase12, caspase3 and cleaved caspase3 expression by Western blot. Statistical analysis was performed using one-way ANOVA followed by Tukey’s multiple comparison test. Each experiment was repeated at least three times. ***p < 0.001 versus control, #p < 0.05, ##p < 0.01, ###p < 0.001 versus Model

LPS treatment increased the levels of IL-6, IL-1β and TNF-α compared with control group (Figure 11A). After rhein treatment, the accumulation of lipid droplets was decreased compared with Model group in a dose-dependent manner (Figure 11B). We also performed a series of in vitro assays so as to measure apoptotic levels and apoptosis-related proteins. Rhein treatment in vitro decreased apoptosis rate and the protein levels of caspase12 and caspase3 (Figure 11C-E).

## Rhein treatment affected inflammation, ERs and autophagy

**Figure 12.**
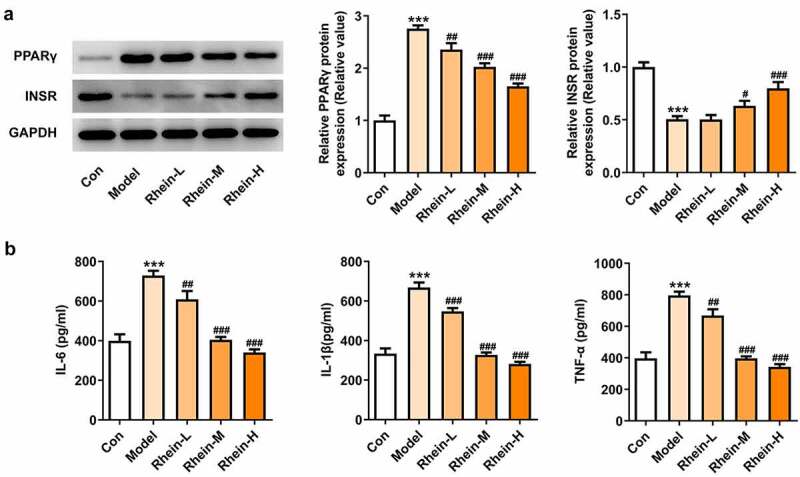
Rhein treatment affected PPARγ and INSR protein levels, and inflammation. The supernatant culturing Raw246.7 cells with LPS stimulation was collected and used to incubate with 3T3-L1 preadipocytes. (a). The expression of PPARγ and INSR. (b). The quantitative analysis of inflammatory factor IL-6, IL-1β and TNF-α. Each experiment was repeated at least three times. ***p < 0.001 versus control. #p < 0.05, ##p < 0.01, ###p < 0.001 versus Model

**Figure 13.**
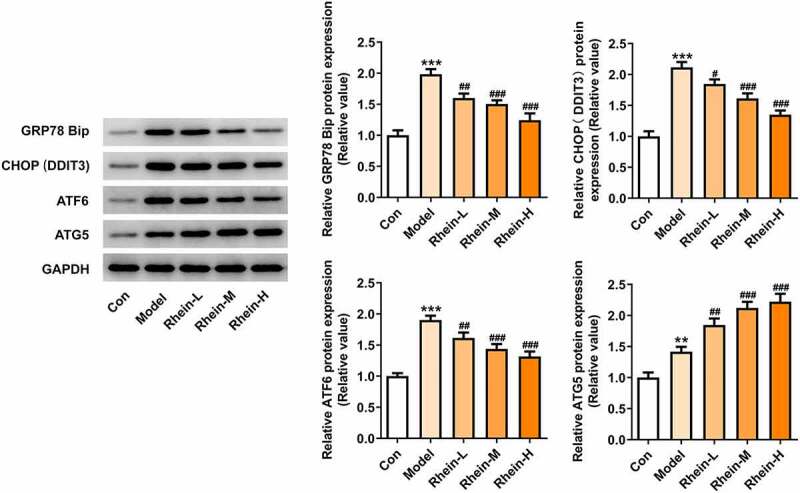
Rhein treatment affected ERs and autophagy of 3T3-L1 cells with inflammatory stimulation. Representative images and quantification of ERs-related proteins and autophagy-related protein in 3T3-L1 cells with inflammatory supernatant induction. The supernatant culturing Raw246.7 cells with LPS stimulation was collected and used to incubate with 3T3-L1 preadipocytes. Each experiment was repeated at least three times. ***p < 0.001 versus control. #p < 0.05, ##p < 0.01, ###p < 0.001 versus Model

**Figure 14.**
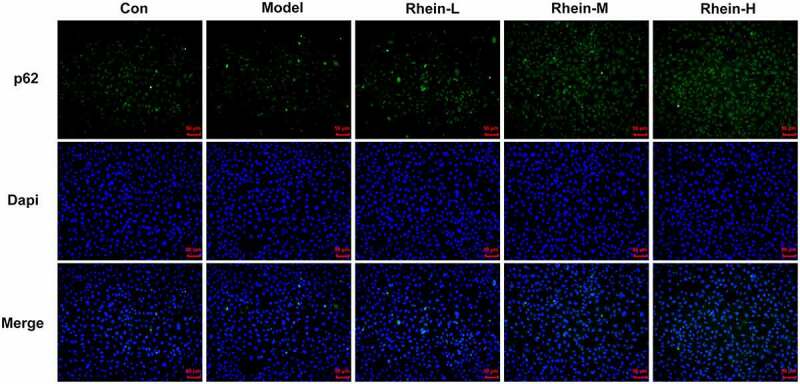
Rhein treatment increased p62 protein levels. Representative images of p62 immunoreactivity (green) in 3T3-L1 cells. The supernatant culturing Raw246.7 cells with LPS stimulation was collected and used to incubate with 3T3-L1 preadipocytes. This experiment was repeated four times. **p < 0.01, ***p < 0.001 versus control. #p < 0.05, ##p < 0.01, ###p < 0.001 versus Model

To determine whether rhein treatment could influence PPARγ, a key gene in adipogenesis, we performed western blot assay in 3T3-L1 cells treated with the supernatant culturing Raw246.7 macrophages. 3T3-L1 cells with inflammatory factors showed a significant increase in PPARγ expression and a decrease in INSR expression, both of which were greatly reversed by rhein (Figure 12A). The levels of IL-6, IL-1β and TNF-α detected by ELISA assay were found to be reduced after rhein treatment compared to those in Model group (Figure 12B). Subsequently, we investigated whether rhein could affect ER stress and autophagy in 3T3-L1 cells induced by inflammatory supernatant. An increase in GRP78, CHOP and ATF6 expressions was specifically observed in Model group as relative to control (Figure 13). Rhein treatment decreased the levels of ERs-related proteins, including GRP78, CHOP and ATF6, and autophagy-related protein ATG5 compared with Model (Figure 13). According to immunofluorescence analysis, we found that the diminished density of p62 was then increased by rhein treatment (Figure 14).

## Discussion

Obesity, a nutritional disorder caused by the accumulation of body fat in certain parts of the body, has adverse effects on health. Many in vitro and animal studies have demonstrated the role of ERs in the pathophysiology of obesity [28–31]. Many studies have demonstrated that ERs activation is involved in HFD-induced obesity [32–34]. ERs is believed to cause ablation in insulin receptors and lead to IR as well as activate NF-κB signaling to produce inflammatory cytokines, thereby inducing inflammation [35].

In the current study, rhein administration could reduce weight gain but had no influence on body length and Lee’s index. Additionally, the insulin, IR, and blood lipid were apparently reduced in rats fed with HFD and rhein, suggesting that rhein could improve metabolic disorder induced by HFD. Then, we found that rhein significantly reduced the levels of IL-6, IL-1β, and TNF-α as well as ameliorated oxidative stress by decreasing ROS and MDA levels and increasing SOD activity in rats with high-fat diet. In parallel, rhein contributed to a reduction in protein levels of caspase12, GRP78, CHOP, and ATF6. Meanwhile, the cell apoptosis in blood vessels and adipose tissue was markedly decreased. Furthermore, increased PPARγ levels and decreased INSR levels caused by high-fat diet were significantly recovered after rhein treatment; therefore, rhein improved IR partly through lessening ERs, oxidative stress, and inflammatory response. A preclinical study showed that ERs inhibition decreased the expression of inflammatory factors, which is closely associated with the inhibited expressions of iRhom2, TACE, TNFR2 and phosphorylated NF-κB, lessened oxidative stress in rats fed with high fat diet as well as decreased lipid accumulation [33]. Therefore, it was possible that the inhibition of ERs by rhein could blunt inflammation and oxidative stress. Intriguingly, these effects of rhein depended on the dose of rhein. It was reported that ATF6 could induce apoptosis via activating CHOP [36]. Therefore, we speculated that rhein reduced apoptosis possibly via decreasing ERs. In vitro, we further explored the mechanism of rhein involved in the 3T3-L1 cells adipogenesis.

As shown in figures, similar effects also appeared in rhein-treated 3T3-L1 cells. We observed that rhein treatment significantly lowered the levels of inflammatory factors, cell apoptosis, GRP78, CHOP and ATF6. Concurrently, the fluorescence intensity of p62 and ATG5 levels were markedly enhanced by rhein treatment, indicating that the attenuation of adipogenesis could in part be attributed to the decreased autophagy by rhein treatment. ERs and autophagy were implicated in the induction of apoptosis and inflammation in mature adipocytes and ERs could precede autophagy [37]. Thus, rhein treatment reduced adipogenesis, inflammation, and apoptosis possibly through regulating ERs and autophagy. After fed with high-fat diet and administrated with rhein, PPARγ levels were decreased and INSR expression was upregulated in peripheral blood and adipose tissue. Furthermore, rhein treatment has reversed the enhanced expression of PPARγ and decreased expression of INSR in 3T3-L1 cells treated with the supernatant culturing Raw246.7 cells after LPS stimulation. PPARγ and INSR are closely associated with IR [38–41]. The inhibition of preadipocytes adipogenesis was considered to be related with the involvement of ERs, PPARγ and INSR.

## Conclusion

The current results evidenced that the potential mechanism accounting for the role of rhein against metabolic disorders induced by high-fat diet is involved in ERs, inflammation, oxidative stress. Understanding the action mechanisms of rhein is of paramount importance, which laid experimental fundation for its clinical use in the therapy of obesity. In addition, it provided us with new sights in understanding the potential molecular mechanisms of rhein.

## Limitation

Lacking of deeper studies about the action mechanism of rhein, by which rhein suppresses adipogenic differentiation needs further supplementation in vitro and vivo. Additionally, the mechanism by which rhubarin affects the endoplasmic reticulum, inflammation, oxidative stress, and the role of rhein in carbohydrate metabolism need further exploration.

## Highlights

1. The beneficial effects of rhein involves in decreased weight gain induced by HFD, and improved serum lipid level, blood glucose and insulin levels.

2. The anti-obesity effects of rhein are associated with ERs, autophagy and inflammation in vivo and vitro.

3. PPARγ and INSR play a vital role in the protective effects of rhein against HFD-induced obesity.
